# Harnessing the Power of Hybrid Light Propagation Model for Three-Dimensional Optical Imaging in Cancer Detection

**DOI:** 10.3389/fonc.2021.750764

**Published:** 2021-09-23

**Authors:** Lin Wang, Wentao Zhu, Ying Zhang, Shangdong Chen, Defu Yang

**Affiliations:** ^1^ School of Computer Science and Engineering, Xi’an University of Technology, Xi’an, China; ^2^ Zhejiang Lab, Research Center for Healthcare Data Science, Hangzhou, China; ^3^ School of Information Sciences and Technology, Northwest University, Xi’an, China; ^4^ Intelligent Information Processing Laboratory, Hangzhou Dianzi University, Hangzhou, China

**Keywords:** hybrid light propagation model, radiosity theory, diffusion approximation, simplified spherical harmonics approximation, optical imaging, cancer detection

## Abstract

Optical imaging is an emerging technology capable of qualitatively and quantitatively observing life processes at the cellular or molecular level and plays a significant role in cancer detection. In particular, to overcome the disadvantages of traditional optical imaging that only two-dimensionally and qualitatively detect biomedical information, the corresponding three-dimensional (3D) imaging technology is intensively explored to provide 3D quantitative information, such as localization and distribution and tumor cell volume. To retrieve these information, light propagation models that reflect the interaction between light and biological tissues are an important prerequisite and basis for 3D optical imaging. This review concentrates on the recent advances in hybrid light propagation models, with particular emphasis on their powerful use for 3D optical imaging in cancer detection. Finally, we prospect the wider application of the hybrid light propagation model and future potential of 3D optical imaging in cancer detection.

## 1 Introduction

Most of the current clinical detection of tumors relies on morphological changes occurring for discrimination, which makes it difficult to see accurately at an early stage. Scientists are working to find a new way to combat this problem. The molecular changes of the tumors are usually earlier than their morphological changes. Optical imaging (OI) has emerged as a strong competitor for early tumor detection due to its ability to observe biological processes at the molecular or cellular level and its high sensitivity, high spatial and temporal resolution, and low cost (1). The optical window from 400 to 1,700 nm is commonly used in OI, and in particular, light in the near infrared II region at 1,000 to 1,700 nm can be used for deep tissue imaging (2–4). Usually, OI is the acquisition of optical signals emitted from the body surface of a living organism, which can reflect the early molecular changes of lesions in the body. Such two-dimensional (2D) planar imaging cannot provide accurate depth and location information of the target and is also difficult to provide accurate quantitative information. By combining the anatomical structure of the organism and optical parameters of biological tissues, the corresponding three-dimensional (3D) imaging technology, called optical tomography (OT), can obtain the spatial localization and distribution, as well as the quantitative information of the targeted probes inside the body from the 2D optical images measured on the body surface (5). This is an important method for quantitative detection of early tumor.

In OT, 3D image reconstruction involves two aspects, namely, the construction of an imaging model that describes the interaction of light with the biological tissues and the development of source reconstruction algorithm for solving the imaging model. Usually, the interaction of light with biological tissues can vary depending on the wavelength of light and the type and characteristics of the tissue. Thus, we need to fully understand and model the various types of interactions between light and biological tissues (6). The propagation mode and process of light in biological tissues directly affect the results of 3D image reconstruction in OT. Building an accurate and fast imaging model, that is, how to describe the propagation of light accurately and fast through biological tissues, is an important prerequisite and foundation for accurate 3D image reconstruction in OT. In the field of OT, the radiative transfer equation (RTE) is usually used to model the process of light propagation in biological tissues (7). However, the RTE can only be solved in a few simple cases. In real biological tissues, it is hard to obtain the solution, which greatly limits its application in OT. In practical applications, various approximation models for RTE are usually used to describe the light propagation process in biological tissues, including the Monte Carlo (MC) simulation (8, 9), diffusion approximation (DA) (5), simplified spherical harmonics approximation (SP_N_) (10), spherical harmonics approximation (P_N_) (11), discrete ordinates approximation (S_N_) (12, 13), and phase approximation (PA) (14). The different approximation models have their own advantages, disadvantages, and scope of application. For example, the MC simulation is regarded as the golden standard for describing the light propagation in biological tissues. However, in practical application, it is difficult to bear the cost of computing time. As the first-order approximation of RTE, DA is the fastest model for describing light propagation in tissues, but its accuracy is limited, especially in the low-scattering region, the high absorption region, and at boundaries. Higher-order approximation models, such as SP_N_, P_N_, S_N_, and PA, can provide a more accurate description of the light propagation process in biological tissues with different optical properties, but similar to the MC simulation, they are more time costly (14–19).

Biological tissues are very complex, with a wide variety of tissue structures and variability in the parameters of optical properties (20). Therefore, it is difficult to describe the propagation of light rapidly and accurately through complex biological tissues using the single approximate model described above. The coupling of different approximation models, and thus constructing a hybrid light propagation model, is a promising solution. Several hybrid light propagation models have been developed to deal with these problems in recent years, including hybrid models for solving non-scattering problems (21–27), hybrid models for solving problems with different scattering characteristics (28–30), and models that can solve both types of problems simultaneously (20). These hybrid models are also applied to OT and enable 3D localization and quantitative detection of tumors *in vivo*, as well as longitudinal monitoring of tumor growth (20, 28, 31). In this review, we concentrate on the recent advances in hybrid light propagation models, including the concept and purpose of the hybrid model, the construction of the models, and their powerful use for 3D optical imaging in cancer detection. Finally, we prospect the wider application of hybrid light propagation models and future potential of 3D optical imaging in cancer detection.

## 2 Light Propagation Models

### 2.1 Light Propagation Models

The propagation behavior of light in biological tissues is complex and diverse, which is related to the properties of light, the structure of biological tissues, and the physical, chemical, and biological properties of biological tissues, including reflection, absorption, scattering, refraction, and transmission. In OI, it is assumed that the scattering of light is completely elastic scattering, that is, scattering only changes the direction of light transmission without changing its frequency. The absorption of light is assumed to be complete absorption. It is well known that the RTE can accurately describe the propagation process of light in biological tissues (32).


(1) $$\frac{1}{c}\frac{\partial L(\vec{r},\hat{s},t)}{\partial t}=-\hat{s}\cdot \nabla L(\vec{r},\hat{s},t)-\mu_{t}L(\vec{r},\hat{s},t)+\mu_{s}\int_{4\pi }L(\vec{r},{\hat{s}}^{'},t)p({\hat{s}}^{'}\cdot \hat{s})d\Omega^{'}+S(\vec{r},\hat{s},t)$$

where $\left(\vec{r},\hat{s},t\right)$ is a coordinate representing spatial position, angular direction, and time; *c* is the light speed in biological tissues; *μ_t_* = *μ_a_* + *μ_s_* is the attenuation coefficient, where *μ_a_* is the absorption coefficient and *μ_s_* is the scattering coefficient; $L(\vec{r},\hat{s},t)$ is the energy in the direction $\hat{s}$ ; $p({\hat{s}}^{'}\cdot \hat{s})$ is the scattering phase function; and $S(\vec{r},\hat{s},t)$ represents the spatial distribution of the light emission source.

As mentioned earlier, the time cost and complexity of the model calculations make the RTE model impractical for application in practical imaging of living organisms. Among the light propagation models based on RTE and its approximation, MC simulation, DA, and SP_N_ are the most frequently chosen in OT.

#### 2.1.1 Monte Carlo Simulation

MC is a random sampling and statistical test method for solving RTE. As an early stochastic modeling technique applied to radiative transfer problem, MC was first introduced into optical transfer problem (8). Wang et al. developed a simulation software (MCML) for optical transmission in multilayer flat media in 1995, which is used widely even today due to its user-friendliness (9). Li et al. and Ren et al. developed the molecular optical simulation environment (MOSE), which can simulate the light transmission in complex media in both two and three dimensions (33, 34).

In the MC method, the radiation of light source is assumed to be photon flow and then discrete into a certain number of photons. The transmission process of many photons in the medium is simulated to solve the optical transmission problem. The transmission process of photons in media is divided into three parts: the generation of photons, the transmission of photons, and the termination of photons. The generation of photons is to determine the initial state of various properties of a single photon according to the shape, energy, and other optical properties of the light source. The properties of photons include wavelength, weight, position, transmission direction, step size, and so on. In the transmission process, photons need to change their own state according to the geometric structure and optical properties of the medium. The information of the medium is deterministic, but the change in photon state is uncertain. This uncertainty in photon transport is the unique characteristic of the MC method. Therefore, using the MC method to solve RTE needs a lot of photon transmission simulation to ensure the accuracy of the results. In the process of photon transmission, due to some reasons, the transmission behavior is terminated, and the transmission process is no longer continued. There are many reasons for photon termination, such as being completely absorbed by the medium, passing through the outermost boundary of the medium and entering the surrounding environment, or being received by the detector.

#### 2.1.2 DA-Based Light Propagation Model

DA is the first-order approximation of RTE, which is derived when the medium in which the light transmission takes place is a highly scattering medium. Because of its low computational complexity and high efficiency, DA is the most widely used light propagation model in OT (35–37). The steady-state form of the DA equation can be expressed as (5):


(2) $$-\nabla \cdot D(\vec{r})\nabla \Phi (\vec{r})+\mu_{a}(\vec{r})\Phi (\vec{r})=S(\vec{r})$$

where $\Phi (\vec{r})$ is the photon flux density at position $\vec{r}$ ; $S(\vec{r})$ is the photon flux density of light emission source; and $D(\vec{r})$ is the diffusion coefficient and defined as $D(\vec{r})={\left(3(\mu_{a}(\vec{r})+(1-g)\mu_{s}(\vec{r}))\right)}^{-1}$ , where *g* is the anisotropy factor.

In OT, the imaging experiment is usually performed in a totally dark environment, so that no photons from the external environment enter. The boundary conditions can be divided into matched boundary conditions without reflection and mismatched boundary conditions with reflection. The refractive index matched boundary indicates that the refractive index of the biological environment on both sides of the boundary is the same. On the contrary, the refractive index of the biological environment represented by the mismatch boundary is different. In OT, the boundary between the biological tissue and environment we encounter has reflective behavior, and the Robin boundary condition is usually used (38).

#### 2.1.3 SP_N_-Based Light Propagation Model

In OT in the near infrared light band, the use of DA to describe the light propagation process is efficient and accurate. However, its accuracy is conditional on the medium through which the light is transmitted having high-scattering properties. Another model that is commonly used in OT is the SP_N_ (39–42). Compared with DA equation, the SP_N_ equation can describe the optical propagation process more accurately and is not limited by the optical properties of biological tissues. As a higher-order approximation to the RTE (18), the SP_N_ has higher accuracy than the lower-order DA, but also brings greater computational complexity and time cost, especially when the order N is higher.

As the accuracy of the SP_N_ model does not improve greatly with increasing the order N, a third- or fifth-order SP_N_ model is usually used in OT. According to a large number of experimental investigations, when N is set as 3, the SP_N_ equation can achieve higher accuracy and computational efficiency. Here, the concrete form of SP_3_ as well as its boundary condition is given:


(3) $$\{\begin{array}{c} -\nabla \cdot \frac{1}{3\mu_{a1}(\vec{r})}\nabla \Phi_{1}(\vec{r})+\mu_{a}(\vec{r})\Phi_{1}(\vec{r})-\frac{2}{3}\mu_{a}(\vec{r})\Phi_{2}(\vec{r})=S(\vec{r})\\ -\nabla \cdot \frac{1}{7\mu_{a3}(\vec{r})}\nabla \Phi_{2}(\vec{r})-\frac{2}{3}\mu_{a}(\vec{r})\Phi_{1}(\vec{r})+\left(\frac{4}{9}\mu_{a}(\vec{r})+\frac{5}{9}\mu_{a2}(\vec{r})\right)\Phi_{2}(\vec{r})=-\frac{2}{3}S(\vec{r}) \end{array}$$

where $\Phi_{1}(\vec{r})$ and $\Phi_{2}(\vec{r})$ are the composite moments related to the flux density at node of $\vec{r}$ ; *μ_ai_* = *μ_a_* + *μ_s_* (1 – *g^i^*)(*i*=1,2,3) are absorption-related coefficients.

### 2.2 Hybrid Models for Solving Non-Scattering Problem

Non-scattering tissues, also called void region, are a kind of special area in an organism, such as gastric cyst, gallbladder, intestine, esophagus, and cerebrospinal fluid. Near infrared optical imaging and OT are increasingly being used to monitor brain oxygenation, hemodynamics, and gastric cancer detection. All these applications have encountered non-scattering problem. It has been shown that the presence of a void region even if it is only 2 mm in size can significantly affect the accuracy of optical tomographic results (43). Therefore, the void problem must be solved in OT. The light propagation in a void region is a different process to that in a scattering medium. In the void region, light is transmitted along a straight line, rather than being a diffuse process. Several methods have been developed to solve such void problem and summarized as follows.

#### 2.2.1 Hybrid Monte-Carlo-Diffusion Model

The MC method is regarded as the golden standard to simulate light propagation in a turbid medium. Thus, the MC method can also be used to process light propagation in void regions. Hayashi et al. proposed a hybrid Monte-Carlo-diffusion method (22, 44), in which the MC method was used to deal with the void problem accurately, and the DA equation was used in the scattering regions. They applied this hybrid model to solve the heterogeneity of the tissues in a head, especially the cerebrospinal fluid (CSF). The hybrid model was verified by comparison with the calculation of the MC method. Results showed that the head model calculated by the hybrid method was in good agreement with the results calculated by MC method, while the results calculated only by DA have obvious errors caused by the effect of the CSF layer. Furthermore, the computation time of the hybrid model is much shorter than that of the MC method.

#### 2.2.2 Hybrid RTE–DA Model

The RTE is the most accurate model for describing the process of light propagation within biological tissues. It can accurately describe light propagation in biological tissues with different scattering properties. In 2005, Tarvainen et al. presented a hybrid model that coupled the RTE and DA, which can solve the problem of light transmission in low-scattering and non-scattering regions (24, 45). In this model, RTE is used as the model of light propagation in the subdomain, in which the assumption of DA is not valid. These subdomains include the proximity of the source, boundary, low-scattering, and non-scattering regions. DA is applied as a forward model to other regions outside the mentioned regions. The two equations are coupled through boundary conditions and solved by the finite element method. The hybrid model was validated with a 2D simulation model, and the results were compared with the RTE, DA, and MC simulation. The results showed that the hybrid RTE–DA model gives almost the same results as the RTE and MC simulation but requires less computing burden. The developed hybrid RTE–DA model can be expressed as:


(4) $$\begin{array}{ll} \begin{array}{cc} \left(\frac{i\varpi }{c}+\hat{s}\cdot \nabla +\mu_{s}+\mu_{a}\right)\phi \left(\vec{r},\hat{s}\right)=\mu_{s}\int_{S^{n-1}}\phi (\vec{r},{\hat{s}}^{\prime })\Theta (\hat{s},{\hat{s}}^{\prime })d{\hat{s}}^{\prime }+S(\vec{r},\hat{s}), & \vec{r}\in \Omega_{RTE} \end{array}\\ \phi (\vec{r},\hat{s})=\left\{ \begin{array}{l} \begin{array}{ccc} \phi_{0}(\vec{r},\hat{s}), & \vec{r}\in \cup_{j}\xi_{j}, & \hat{s}\cdot \hat{n}<0 \end{array}\\ \begin{array}{ccc} 0, & \vec{r}\in \partial \Omega_{RTE,out}\cup_{j}\xi_{j}, & \hat{s}\cdot \hat{n}<0 \end{array} \end{array}\right.\\ \begin{array}{cc} \phi (\vec{r},\hat{s})=\frac{1}{\left|S^{n-1}\right|}\Phi (\vec{r})-\frac{n}{\left|S^{n-1}\right|}\hat{s}\cdot \left(\kappa \nabla \Phi \left(\vec{r}\right)\right), & \vec{r}\in \Gamma \end{array}\\ -\nabla \cdot \kappa \nabla \Phi (\vec{r})+\mu_{a}\Phi (\vec{r})+\frac{i\varpi }{c}\Phi (\vec{r})=S_{0}(\vec{r}),\vec{r}\in \Omega_{DA} & \begin{array}{cc} \Phi (\vec{r})=\int_{S^{n-1}}\phi (\vec{r},\hat{s})d\hat{s}, & \vec{r}\in \Gamma \end{array} \end{array}$$

Here, all the parameters that do not appear can be found in (24) for detailed description.

Subsequently, Gorpas et al. first implemented the hybrid RTE–DA model in 3D and applied it to fluorescence molecular imaging (FMI) in 2010 (46). In their study, a hyperellipsoid model was used to mimic the tumor lesion. Similarly, the hybrid RTE–DA model was also compared with the RTE and DA models. The experimental results showed that the hybrid RTE–DA model is much closer to RTE, even in the DA subdomain. Although the hybrid RTE–DA model does not present computational times close to the rapid DA, it is still faster than the RTE forward solver, where the RTE–DA saved about 50% of computational time cost compared with the RTE forward solver in experiments (24). The authors analyzed that most of the computational time required for the hybrid RTE–DA model is spent solving the phase function. With the phase function calculated in advance, the hybrid model can achieve convergence at the same time as the DA calculation. In 2012, the hybrid RTE–DA model was applied to the digital mouse for the first time, which is modified to form a forward solver for dual-coupled FMI (25). The results were compared with those of the MC method and the RTE and showed that the proposed forward solver can approximate the RTE and MC method with an accuracy better than 95%, while the accuracy of the DA is approximately 10% lower.

#### 2.2.3 Incorporating Radiosity Equation in the Hybrid Model

The radiosity equation can also be applied to describe light propagation in the void region. Thus, it can be incorporated with the DA or higher-order approximation of RTE to construct the hybrid light propagation model. In such hybrid model, the DA or higher-order approximation of RTE is used to model the light propagation in scattering medium, and the radiosity equation is employed to characterize light propagation in the void region.

In 1996, Arridge et al. proposed a hybrid method specially designed to deal with zero scattering problem (21) and a predecessor to the hybrid radiosity-diffusion model (HRDM) in which they used DA for the scattering area and applied the linear light propagation properties to the cavity region. This hybrid model was validated with a simple flat plate model by comparing with the MC simulation and experimental results. The experimental results show that the existence of a clear layer has a significant effect on the light distribution. This effect can be accurately simulated by Monte Carlo, but not by diffusion approximation. The experimental results of the proposed hybrid model are consistent with those of Monte Carlo simulation.

In 2000, the HRDM was formally proposed and combined with the finite element method for numerical calculation, which enhanced the geometrical flexibility of the hybrid model (47). In their work, all aspects of the implementation were explained and how to extend the analysis to frequency domain and time domain problems was given. Immediately afterwards, they explored the influence of void regions on diffuse optical tomography and completed a 3D expansion of this hybrid model (48). To our knowledge, this is the first assessment of optical tomography in a 3D geometry involving voids. In 2012, Chen et al. extended the HRDM to a 3D digital mouse model and applied it to bioluminescence tomography (BLT) (31). The advantages of the BLT based on this hybrid model for the detection of cavity-based tumors were confirmed by comparison with the DA. The concise form of the HRDM can be expressed as (31):


(5) $$-\nabla \cdot [D(\vec{r})\nabla \Phi (\vec{r})]+\mu_{a}(\vec{r})\;\Phi \;(\vec{r})=S(\vec{r})+\int_{B}\frac{1}{\pi }J_{n}\left({\vec{r}}^{\prime }\right)G\left({\vec{r}}^{\prime },\vec{r}\right)dB$$

where *B* is the interface between the scattering and void regions, $J_{n}({\vec{r}}^{\prime })$ is the light flux rate formed at the interface *B* and directed toward the interior of the cavity, and $G({\vec{r}}^{\prime },\vec{r})$ is the photon transfer function between different points of the interface *B*.

As previously described, DA has high accuracy only in the high-scattering region and poor accuracy at low scattering, high absorption, or at the source and boundary. In living organisms, low-scattering, high absorption regions are always present alongside high-scattering and no scattering regions. In this case, HRDM is not suitable for whole-body imaging of small animals. To solve this problem, Yang et al. developed a novel hybrid light propagation model that couples the SP_N_ with the radiosity equation (HSRM) (26). Similarly, the radiosity equation was used to characterize light propagation in void regions. The difference is that the SP_N_ was employed to handle the propagation of light through a scattering medium. The hybrid model was also validated with the digital mouse model, and relevant results showed that it provided high performance for light propagation in media with non-scattering, low-scattering, high-scattering, and high absorption heterogeneities. The HSRM was also selected as the forward model of the BLT for gastric cancer detection (27), and the results proved that the HSRM-based 3D optical imaging can significantly improve the accuracy of the HRDM-based one.

#### 2.2.4 Other Models

In 2013, Lehtikangas et al. continued to study hybrid models and proposed a method for developing a hybrid model by combining forward-peaked scattering approximations of the RTE and diffusion approximation (49). In this model, the computational domain was divided into two subdomains. The Fokker–Planck equation or the Fokker–Planck–Eddington equation was used in the subdomains in which the DA is not valid, such as close to the source and boundary, in low-scattering regions, and so on. The DA is used in high-scattering regions. The two kinds of equations are coupled at the interfaces of the subdomains with boundary conditions and solved simultaneously using the finite element method. The proposed methods were verified with a realistic head geometry by comparing with the RTE, DA, and the hybrid RTE–DA model. The results show that the proposed models can be used to describe light propagation in heterogeneous tissues, also with low-scattering regions such as the cerebrospinal fluid in the brain, with almost the same accuracy as the RTE but with reduced computational load.

### 2.3 Hybrid Models for Solving Different Scattering Regions

In a biological body, biological tissues have very different tissue optical properties, including tissues with different scattering properties, tissues with different absorption properties, and being very dependent on both the type of tissue and the wavelength of light. In order to find a solution for light propagation in N-layered turbid media with different scattering properties, the hybrid diffusion-P_3_ equation was developed for an N-layered finite or infinite turbid medium in the steady-state domain for one point source using the extrapolated boundary condition (50, 51). The main difference between the DA and the hybrid diffusion-P_3_ equation is the diffusion coefficient used in the DA and the asymptotic diffusion coefficient of the hybrid diffusion-P_3_ equation. With the decrease of the absorption coefficient, the values of these two coefficients tend to be the same, and the solutions of the DA and the hybrid diffusion-P_3_ equation are basically the same. The corresponding results concluded that the hybrid diffusion-P_3_ equation is closer to the Monte Carlo simulation than the DA, so that the hybrid diffusion-P_3_ equation can replace the DA for light propagation in turbid media for a wide range of absorption coefficients.

To avoid the limitations of the SP_N_ and DA in describing light propagation in biological tissues, Chen et al. proposed a hybrid SP_N_ with DA (HSDE) model, in which the DA was selected for describing light propagation in high-scattering tissues and the SP_N_ was used for other scattering tissues, including low scattering, high absorption, and so on (29, 52). In their study, the living body was first segmented into several major organs, and these organs were classified into high-scattering tissues and other scattering tissues. They established a boundary coupling condition to combine the SP_N_ and DA to finally form the hybrid model. The performance of the hybrid model was validated with both regular geometries and digital mouse model, and results revealed that the HSDE model makes full use of the advantages of SP_N_ and DE in terms of accuracy and efficiency. Subsequently, Chen et al. and Wang et al. applied the HSDE model as the forward imaging model for fluorescence molecular tomography (28), multispectral Cerenkov luminescence tomography (53), and X-ray luminescence computed tomography (54), respectively. The concise form of the HSDE model can be expressed as (29):


(6) $$-\nabla \cdot C_{k,\nabla \Phi_{1}}(\vec{r})\nabla \Phi_{1}(\vec{r})-\nabla \cdot C_{k,\nabla \Phi_{2}}(\vec{r})\nabla \Phi_{2}(\vec{r})+C_{k,\nabla \Phi_{1}}(\vec{r})\Phi_{1}(\vec{r})+C_{k,\nabla \Phi_{2}}(\vec{r})\Phi_{2}(\vec{r})=C_{k,S}(\vec{r})S(\vec{r})$$

Here, the relevant parameters can be found in detail in (29).

### 2.4 Adaptively Alternative Light Propagation Model

In whole-body imaging of living small animals, biological tissues have complex tissue specificities, including the heterogeneity in organ structure and diversity in tissue optical properties. Different biological tissues respond differently to light at the same wavelength, and the same biological tissue also responds differently to light at different wavelengths. There is therefore an urgent need for an imaging model in OT that can accurately and efficiently handle light propagation in biological tissues in this complex situation. To address this problem, Chen et al. proposed an adaptively alternative light transport model and its corresponding optical 3D imaging method for the detection of *in situ* gastric cancer (20). In this hybrid model, the authors used the DA to describe light propagation in the high-scattering tissues, the SP_N_ for the low-scattering or high absorption tissues, and the radiosity equation for the void regions. These three equations were coupled by constructing corresponding boundary conditions at different types of biological tissue boundaries. The coupled unified form was also termed as the hybrid SP_N_–DA–radiosity (HSDR) model. The so-called adaptation refers to the selection of the most appropriate equation to describe the light transport process in biological tissues. The HSDR model makes full use of the specificity of the biological tissues, including both the anatomical structure and optical properties, which facilitates to improve both the quality and efficiency of the reconstructed images. The concise form of the HSDR model can be expressed as (20):


(7) $$\begin{array}{l} \begin{array}{cc} \begin{array}{l} \sigma (\vec{r})\left\{-\nabla \cdot \xi_{i,\nabla \varphi_{i}}^{}\nabla \varphi_{i}(\vec{r})+\sum_{j=1}^{(N+1)/2}\xi_{i,\varphi_{j}}^{}\varphi_{j}(\vec{r})\right\}\\ +\delta (i-1)(1-\sigma (\vec{r}))\left\{-\nabla \cdot \frac{1}{3(\mu_{a}+(1-g)\mu_{s})}\nabla \varphi_{0}(\vec{r})+\mu_{a}\varphi_{0}(\vec{r})\right\}\\ =\sigma (\vec{r})\left\{\xi_{i,S}^{}S(\vec{r})+\sum_{j=1}^{(N+1)/2}\int_{B_{v}}h(\vec{r},{\vec{r}}^{\prime })\frac{\xi_{i,S}^{}\beta_{j}\varphi_{j}({\vec{r}}^{\prime })\cos\theta \cos\theta^{\prime }}{\pi |\vec{r}-{\vec{r}}^{\prime }{|}^{2}}e^{-\mu_{av}|\vec{r}-{\vec{r}}^{\prime }|}dB\right\}\\ +\delta (i-1)(1-\sigma (\vec{r}))\left\{S(\vec{r})+\int_{B_{v}}h(\vec{r},r^{\prime })\frac{\beta_{0}\varphi_{0}(r^{\prime })\cos\theta \cos\theta^{\prime }}{\pi |\vec{r}-r^{\prime }{|}^{2}}e^{-\mu_{av}|\vec{r}-{\vec{r}}^{\prime }|}dB\right\},\; \end{array} & \vec{r}\in \Omega_{hs}\cup \Omega_{hs}\cup B_{v},{\vec{r}}^{\prime }\in B_{v} \end{array}\\ \begin{array}{cc} \begin{array}{l} \sigma (\vec{r})\left\{\sum_{j=1}^{(N+1)/2}\xi_{i,\nabla \varphi_{j}}^{b}\nu \cdot \nabla \varphi_{j}(\vec{r})-\sum_{j=1}^{(N+1)/2}\xi_{i,\varphi_{j}}^{b}\varphi_{j}(\vec{r})\right\}\\ =\delta (i-1)(1-\sigma (\vec{r}))\left\{\frac{1+B_{1}}{3(\mu_{a}+(1-g)\mu_{s})}\nu \cdot \nabla \varphi_{0}(\vec{r})-(\frac{1}{2}+A_{1})\varphi_{0}(\vec{r})\right\},\; \end{array} & \;\vec{r}\in \partial \Omega \cup B_{v}\; \end{array}\\ \begin{array}{cc} \varphi_{1}(\vec{r})=\varphi_{0}(\vec{r}),\;\varphi_{2}(\vec{r})=0,\;\cdots \cdots ,\;\varphi_{N}(\vec{r})=0\;, & \vec{r}\in B_{hl} \end{array} \end{array}$$

Here, the relevant parameters can be found in detail in (20).

## 3 Hybrid Light Propagation Model-Based Optical Tomography

Using numerical discretization methods, the above hybrid models can be transformed into the form of a matrix equation describing the relationship between the internal signal emission source and the light flux density on the surface of the body:


(8) AS=B

Here, A is the system matrix determined from the mesh node distribution and the optical properties information, S is the vector constructed by the internal signal emission source, and B is the photon density measured on the discretized boundary nodes.

Optical tomography is the solution to the signal emission source S based on the measured light flux density B. The solution of Eq (8). is of highly ill-posedness due to the severely deficient nature of the measurement data and is usually solved by transforming it into an objective optimization problem. The objective optimization problem can be written as a minimization problem as follows:


(9) min|AS−B|

The minimization problem of Eq (9). can be solved by many iterative optimization algorithms.

## 4 Applications of the Hybrid Models in Cancer Detection

The light propagation model is the basis and prerequisite for the 3D reconstruction of OT and directly affects the accuracy and efficiency of OT. When applying OT to tumor detection, the site where the tumor is located needs to be considered. Tumors at different sites or organs may have different requirements for the light propagation model to be used for OT. For example, if we want to detect gastric cancer with cavity characteristic, or liver cancer with low-scattering characteristic, the effect of the void or low-scattering region on light propagation should be considered when constructing the light transport model. In this section, we focus on summarizing the applications of the hybrid light propagation models to tumor detection.

### 4.1 Applications of the Hybrid Models in Gastric Cancer Detection

There are two challenging problems encountered in 3D optical imaging of *in situ* gastric cancer. Firstly, the stomach is a cavity-like organ and it has been shown that when the cavity size reaches 2 mm it has a large impact on the results of OT (43). Secondly, the stomach is spatially encapsulated by organs such as the muscle and liver, which have completely different scattering properties. For example, at the wavelengths commonly used in OT, muscle is a high-scattering tissue, while liver is a low-scattering tissue. Therefore, for the detection of *in situ* gastric cancer using OT technology, a hybrid light propagation model that can handle the cavity problem is needed to ensure tumor detection accuracy.

**Figure 1:**
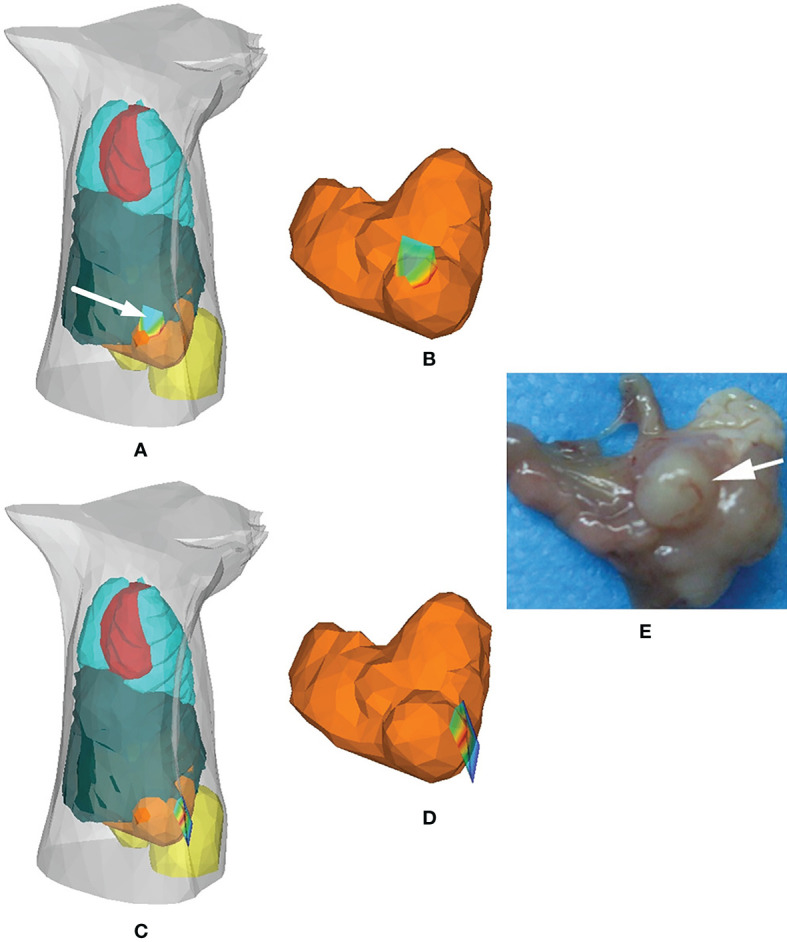
Application of the hybrid radiosity-diffusion model in the detection of *in situ* gastric cancer. **(A, B)** Results of the HRDM-based BLT, **(C, D)** those of the DE-based BLT, and **(E)** the necropsy observation of the tumor lesion. Adapted with permission from (31).

In 2012, Chen et al. compared the performance of HRDM and DA in solving the cavity problem in BLT and applied HRDM to the detection of *in situ* gastric cancer, as shown in **Figure 1**. The results showed that the distribution of tumor cells reconstructed by the HRDM-based BLT was more consistent with those observed at autopsy than the DA-based approach (31). To further address the effect of the presence of low-scattering tissue around the stomach on HRDM, Chen et al. subsequently applied the HRSE to bioluminescence tomographic detection of gastric cancer *in situ* (27). Both the localization and distribution of tumor cells obtained by the HRSE-based BLT were better than those of the HRDM, revealing the applicability and superiority of the HRSE in the application of gastric cancer detection.

**Figure 2:**
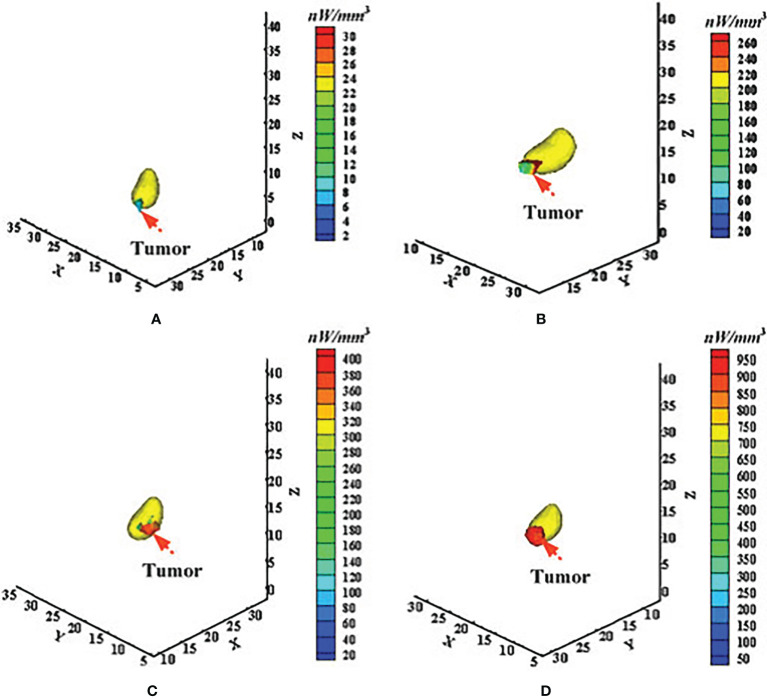
Application of the adaptively alternative light transport model in longitudinal and quantitative monitoring of *in situ* gastric cancer. **(A–D)** Correspond to the time points of 2, 11, 21, and after 28 days of tumor formation. Adapted with permission from (20).

In 2016, Chen et al. applied the adaptively alternative light transport model, also called the HSDR model, to BLT for longitudinal and quantitative monitoring of gastric cancer in live animal (20). Approximately 5 × 10^6^ SGC7901-Luc-GFP cells were injected into the stomach wall of mice to construct *in situ* gastric cancer-bearing mouse models. At the points of 2, 11, 21, and 28-plus days after injection of tumor cells, the optical images were acquired from the body surface and the tumor information was retrieved using the HSDR-based BLT method. **Figure 2** presents the corresponding reconstructed results. The results showed that the reconstructed elements localized the tumor lesion well. Both the reconstructed tumor volume and power density increased progressively over time and were consistent with the measured tumor volume growth trend.

### 4.2 Applications of the Hybrid Models in Liver Cancer Detection

**Figure 3:**
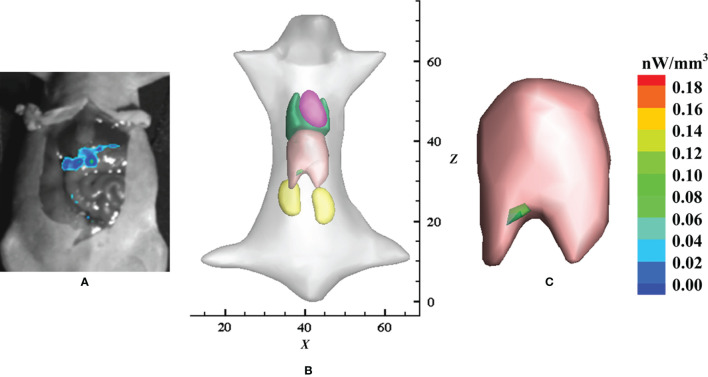
Application of the HSDE model in the detection of *in situ* liver cancer. **(A)** Laparotomy results with the liver exposed and imaged using bioluminescence imaging, **(B)** reconstructed result by the HSDE-based fluorescence tomography, and **(C)** relevant local enlarged image. Adapted with permission from (28).

Very similar to the stomach which has special characteristics, the liver also has its own special characteristics, including the fact that it is usually considered a low-scattering tissue and is surrounded by high-scattering tissues such as muscle, kidney, and heart. This leads to a breakdown of the DA-based OT as well as a heavy computational burden for the SP_N_-based OT. Chen et al. applied the HSDE model to fluorescence tomography for *in situ* detection of liver cancer in living animal (28). The localization and distribution of inoculated HCC-LM3-fLuc-GFP cells were well reconstructed by using the HSDE-based fluorescence tomography (**Figure 3**). The location of the reconstructed tumor cells was of good consistency with the dissected image of bioluminescence imaging, demonstrating the HSDE model has great application potential for *in situ* detection of liver cancer.

## 5 Conclusion and Perspective

We present above an account of recent advances on the hybrid light propagation models, with particular emphasis on their powerful use for 3D optical imaging in cancer detection. An accurate and efficient light propagation model is the core task and premise for building accurate and fast 3D optical imaging methods and technologies. With further development of the light propagation model, and in particular the integration with hybrid models into OT, we believe that 3D optical imaging would be a powerful tool with a great potential in the quantitative detection and longitudinal monitoring of *in situ* cancer. For example, with a more accurate forward model, optical imaging can achieve a 0.3-mm deviation for localization and 6.5% quantitative deviation for energy power of a lesion (55). We anticipate some discussions in the future development of the hybrid model as well as its applications in OT, which provides perspective as well as challenges for researchers.

In order to be widely used in OT for the application of cancer detection, hybrid light propagation models need to fuse the advantages of both accuracy and efficiency. The future development direction of the hybrid model is to further improve calculation accuracy and speed. This can be worked on in two aspects. Firstly, a new hybrid light propagation model can be constructed that guarantees the accuracy and speed by using higher-order approximations of RTE and the acceleration algorithms. The first problem to be faced in constructing a hybrid light propagation model is to establish the coupling between different equations. This coupling relationship enables the conversion of different physical quantities between equations. How to ensure the accuracy and efficiency of the coupling between the higher-order approximation and the lower order approximation of the RTE is also a problem being addressed in the study of hybrid light propagation models. Secondly, a more accurate tissue classification method can be established. The individual differences of optical properties and the errors in tissue segmentation would affect the accuracy of tissue classification, which in turn affects the accuracy of the hybrid light propagation model and the accuracy of the 3D optical imaging. In addition, a deep learning framework, which has recently emerged in OT, can be incorporated into the construction of the hybrid light propagation model to improve accuracy and computational burden (56–59).

Another promising direction is the application of hybrid light propagation models. Currently, there are many studies on the construction of hybrid models, and various hybrid models have been proposed. However, relatively few preclinical applications based on these hybrid models have been carried out. This is most likely due to the complexity and efficiency of existing hybrid light propagation models. By providing 3D quantitative information, such as localization, distribution, and volume of the targeted probe, 3D optical imaging has become an important tool in the field of biomedical science. Because of having the ability to achieve an optimal compromise between accuracy and efficiency, hybrid light propagation models and corresponding 3D optical imaging techniques offer significant advantages in preclinical whole-body imaging of small animals, including quantitative detection and longitudinal monitoring of *in situ* tumor and dynamic monitoring and assessment of drug metabolism *in vivo*. In addition to cancer detection, hybrid light propagation models can be used to address other applications, such as the cerebrospinal fluid-filled ventricles in the brain (44, 47).

## Author Contributions

LW: conceptualization and writing—original draft. WZ and YZ: reviewing. SC and DY: writing, reviewing, and editing. All authors contributed to the article and approved the submitted version.

## Funding

This work was supported by the National Natural Science Foundation of China (Grant Nos. 61801157, 62101439, and 62001425).

## Conflict of Interest

The authors declare that the research was conducted in the absence of any commercial or financial relationships that could be construed as a potential conflict of interest.

## Publisher’s Note

All claims expressed in this article are solely those of the authors and do not necessarily represent those of their affiliated organizations, or those of the publisher, the editors and the reviewers. Any product that may be evaluated in this article, or claim that may be made by its manufacturer, is not guaranteed or endorsed by the publisher.

## References

[1] Alexandrakis G Rannou FR Chatziioannou AF. Tomographic Bioluminescence Imaging by Use of a Combined Optical-PET (OPET) System: A Computer Simulation Feasibility Study. Phys Med Biol (2005) 50(17):4225. doi: 10.1088/0031-9155/50/17/021 16177541 PMC1317109

[2] Phillips WT Klipper R Goins B. Use of 99mtc-Labeled Liposomes Encapsulating Blue Dye for Identification of the Sentinel Lymph Node. J Nucl Med (2001) 42(3):446–51. 11337521

[3] Tichauer KM Samkoe KS Gunn JR Kanick SC Hoopes PJ Barth RJ. Microscopic Lymph Node Tumor Burden Quantified by Macroscopic Dual-Tracer Molecular Imaging. Nat Med (2014) 20(11):1348–53. doi: 10.1038/nm.3732 PMC4224611 25344739

[4] Hu Z Fang C Li B Zhang Z Cao C Cai M. First-In-Human Liver-Tumour Surgery Guided by Multispectral Fluorescence Imaging in the Visible and Near-Infrared-I/II Windows. Nat BioMed Eng (2020) 4(3):259–71. doi: 10.1038/s41551-019-0494-0 31873212

[5] Cong W Wang G Kumar D Liu Y Jiang M Wang LV. Practical Reconstruction Method for Bioluminescence Tomography. Opt Express (2005) 13(18):6756–71. doi: 10.1364/opex.13.006756 19498692

[6] Periyasamy V Pramanik M. Advances in Monte Carlo Simulation for Light Propagation in Tissue. IEEE Rev BioMed Eng (2017) 10:122–35. doi: 10.1109/RBME.2017.2739801 28816674

[7] Ishimaru A. Diffusion of Light in Turbid Material. Appl Optics (1989) 28(12):2210–5. doi: 10.1364/AO.28.002210 20555501

[8] Wilson BC. Adam G. A Monte Carlo Model for the Absorption and Flux Distributions of Light in Tissue. Med Phys (1983) 10(6):824–30. doi: 10.1118/1.595361 6656695

[9] Wang L Jacques SL Zheng L. MCML—Monte Carlo Modeling of Light Transport in Multi-Layered Tissues. Comput Meth Programs BioMed (1995) 47(2):131–46. doi: 10.1016/0169-2607(95)01640-f 7587160

[10] Liu K Lu Y Tian J Qin C Yang X Zhu S. Evaluation of the Simplified Spherical Harmonics Approximation in Bioluminescence Tomography Through Heterogeneous Mouse Models. Opt Express (2010) 18(20):20988–1002. doi: 10.1364/OE.18.020988 20940994

[11] Wright S Schweiger M Arridge S. Reconstruction in Optical Tomography Using the PN Approximations. Meas Sci Technol (2006) 18(1):79. doi: 10.1088/0957-0233/18/1/010

[12] Peng K Gao X Qu X Ren N Chen X He X. Graphics Processing Unit Parallel Accelerated Solution of the Discrete Ordinates for Photon Transport in Biological Tissues. Appl Optics (2011) 50(21):3808–23. doi: 10.1364/AO.50.003808 21772362

[13] Yuan Z Hu X-H Jiang H. A Higher Order Diffusion Model for Three-Dimensional Photon Migration and Image Reconstruction in Optical Tomography. Phys Med Biol (2008) 54(1):65. doi: 10.1088/0031-9155/54/1/005 19060361

[14] Cong W Cong A Shen H Liu Y Wang G. Flux Vector Formulation for Photon Propagation in the Biological Tissue. Opt Lett (2007) 32(19):2837–9. doi: 10.1364/ol.32.002837 17909590

[15] De Oliveira C. An Arbitrary Geometry Finite Element Method for Multigroup Neutron Transport With Anisotropic Scattering. Prog Nucl Energy (1986) 18(1-2):227–36. doi: 10.1016/0149-1970(86)90029-6

[16] Jiang H. Optical Image Reconstruction Based on the Third-Order Diffusion Equations. Opt Express (1999) 4(8):241–6. doi: 10.1364/oe.4.000241 19396281

[17] Aydin E De Oliveira C Goddard A. A Comparison Between Transport and Diffusion Calculations Using a Finite Element-Spherical Harmonics Radiation Transport Method. Med Phys (2002) 29(9):2013–23. doi: 10.1118/1.1500404 12349922

[18] Klose AD Larsen EW. Light Transport in Biological Tissue Based on the Simplified Spherical Harmonics Equations. J Comput Phys (2006) 220(1):441–70. doi: 10.1016/j.jcp.2006.07.007

[19] Aydin E. Three-Dimensional Photon Migration Through Voidlike Regions and Channels. Appl Optics (2007) 46(34):8272–7. doi: 10.1364/ao.46.008272 18059668

[20] Chen X Yang D Sun F Cao X Liang J. Adaptively Alternative Light-Transport-Model-Based Three-Dimensional Optical Imaging for Longitudinal and Quantitative Monitoring of Gastric Cancer in Live Animal. IEEE Trans BioMed Eng (2016) 63(10):2095–107. doi: 10.1109/TBME.2015.2510369 26700857

[21] Firbank M Arridge SR Schweiger M Delpy DT. An Investigation of Light Transport Through Scattering Bodies With Non-Scattering Regions. Phys Med Biol (1996) 41(4):767–83. doi: 10.1088/0031-9155/41/4/012 8730669

[22] Hayashi T Kashio Y Okada E. Hybrid Monte Carlo-Diffusion Method for Light Propagation in Tissue With a Low-Scattering Region. Appl Optics (2003) 42(16):2888–96. doi: 10.1364/ao.42.002888 12790437

[23] Lee JH Kim S Kim YT. Modeling of Diffuse-Diffuse Photon Coupling *via* a Nonscattering Region: A Comparative Study. Appl Optics (2004) 43(18):3640–55. doi: 10.1364/ao.43.003640 15218604

[24] Tarvainen T Vauhkonen M Kolehmainen V Arridge SR Kaipio JP. Coupled Radiative Transfer Equation and Diffusion Approximation Model for Photon Migration in Turbid Medium With Low-Scattering and Non-Scattering Regions. Phys Med Biol (2005) 50(20):4913–30. doi: 10.1088/0031-9155/50/20/011 16204880

[25] Gorpas DS Andersson-Engels S. Evaluation of a Radiative Transfer Equation and Diffusion Approximation Hybrid Forward Solver for Fluorescence Molecular Imaging. J BioMed Opt (2012) 17(12):126010. doi: 10.1117/1.JBO.17.12.126010 23208221

[26] Yang D Chen X Peng Z Wang X Ripoll J Wang J. Light Transport in Turbid Media With non-Scattering, Low-Scattering and High Absorption Heterogeneities Based on Hybrid Simplified Spherical Harmonics With Radiosity Model. BioMed Opt Express (2013) 4(10):2209–23. doi: 10.1364/BOE.4.002209 PMC3799679 24156077

[27] Chen X Zhang Q Yang D Liang J. Hybrid Radiosity-SP3 Equation Based Bioluminescence Tomography Reconstruction for Turbid Medium With Low-and non-Scattering Regions. J Appl Phys (2014) 115(2):024702. doi: 10.1063/1.4862166

[28] Chen X Sun F Yang D Liang J. Coupled Third-Order Simplified Spherical Harmonics and Diffusion Equation–Based Fluorescence Tomographic Imaging of Liver Cancer. J BioMed Opt (2015) 20(9):90502. doi: 10.1117/1.JBO.20.9.090502 26385654

[29] Chen X Sun F Yang D Ren S Zhang Q Liang J. Hybrid Simplified Spherical Harmonics With Diffusion Equation for Light Propagation in Tissues. Phys Med Biol (2015) 60(16):6305. doi: 10.1088/0031-9155/60/16/6305 26237074

[30] Wang L Cao X Ren Q Chen X He X. Hybrid Model Based Unified Scheme for Endoscopic Cerenkov and Radio-Luminescence Tomography: Simulation Demonstration. J Appl Phys (2018) 123(18):184701. doi: 10.1063/1.5026749

[31] Chen X Yang D Qu X Liang J Tian J Hu H. Comparisons of Hybrid Radiosity-Diffusion Model and Diffusion Equation for Bioluminescence Tomography in Cavity Cancer Detection. J BioMed Opt (2012) 17(6):66015. doi: 10.1117/1.JBO.17.6.066015 22734771

[32] Wang LV Wu H-i. Biomedical Optics: Principles and Imaging. John Wiley & Sons (2012), ISBN: 9780470177006 0470177004.

[33] Li H Tian J Zhu F Cong W Wang LV Hoffman EA. A Mouse Optical Simulation Environment (MOSE) to Investigate Bioluminescent Phenomena in the Living Mouse With the Monte Carlo Method1. Acad Radiol (2004) 11(9):1029–38. doi: 10.1016/j.acra.2004.05.021 15350584

[34] Ren S Chen X Wang H Qu X Wang G Liang J. Molecular Optical Simulation Environment (MOSE): A Platform for the Simulation of Light Propagation in Turbid Media. PloS One (2013) 8(4):e61304. doi: 10.1371/journal.pone.0061304 23577215 PMC3620115

[35] Gibson AP Hebden JC Arridge SR. Recent Advances in Diffuse Optical Imaging. Phys Med Biol (2005) 50(4):R1. doi: 10.1088/0031-9155/50/4/r01 15773619

[36] Ntziachristos V Ripoll J Wang LV Weissleder R. Looking and Listening to Light: The Evolution of Whole-Body Photonic Imaging. Nat Biotechnol (2005) 23(3):313–20. doi: 10.1038/nbt1074 15765087

[37] Hu Z Liang J Yang W Fan W Li C Ma X. Experimental Cerenkov Luminescence Tomography of the Mouse Model With SPECT Imaging Validation. Opt Express (2010) 18(24):24441–50. doi: 10.1364/OE.18.024441 21164791

[38] Schweiger M Arridge S Hiraoka M Delpy D. The Finite Element Method for the Propagation of Light in Scattering Media: Boundary and Source Conditions. Med Phys (1995) 22(11):1779–92. doi: 10.1118/1.597634 8587533

[39] Chu M Dehghani H. Image Reconstruction in Diffuse Optical Tomography Based on Simplified Spherical Harmonics Approximation. Opt Express (2009) 17(26):24208–23. doi: 10.1364/OE.17.024208 20052132

[40] Liemert A Kienle A. Analytical Solutions of the Simplified Spherical Harmonics Equations. Opt Lett (2010) 35(20):3507–9. doi: 10.1364/OL.35.003507 20967115

[41] Liu H Yang X Song T Bao C Shi L Hu Z. Multispectral Hybrid Cerenkov Luminescence Tomography Based on the Finite Element SPn Method. J BioMed Opt (2015) 20(8):86007. doi: 10.1117/1.JBO.20.8.086007 26271053

[42] Gao Y Ma X Kang F Yang W Liu Y Wang Z. Enhanced Cerenkov Luminescence Tomography Analysis Based on Y 2 O 3: Eu 3+ Rare Earth Oxide Nanoparticles. BioMed Opt Express (2018) 9(12):6091–102. doi: 10.1364/BOE.9.006091 PMC6491000 31065415

[43] Yang D Chen X Ren S Qu X Tian J Liang J. Influence Investigation of a Void Region on Modeling Light Propagation in a Heterogeneous Medium. Appl Optics (2013) 52(3):400–8. doi: 10.1364/AO.52.000400 23338186

[44] Ogoshi Y Okada E. Analysis of Light Propagation in a Realistic Head Model by a Hybrid Method for Optical Brain Function Measurement. Opt Rev (2005) 12(3):264–9. doi: 10.1007/s10043-005-0264-y

[45] Tarvainen T Vauhkonen M Kolehmainen V Kaipio JP. Hybrid Radiative-Transfer–Diffusion Model for Optical Tomography. Appl Optics (2005) 44(6):876–86. doi: 10.1364/ao.44.000876 15751677

[46] Gorpas D Yova D Politopoulos K. A Three-Dimensional Finite Elements Approach for the Coupled Radiative Transfer Equation and Diffusion Approximation Modeling in Fluorescence Imaging. J Quant Spectrosc Radiat Transf (2010) 111(4):553–68. doi: 10.1016/j.jqsrt.2009.11.006

[47] Arridge SR Dehghani H Schweiger M Okada E. The Finite Element Model for the Propagation of Light in Scattering Media: A Direct Method for Domains With Nonscattering Regions. Med Phys (2000) 27(1):252–64. doi: 10.1118/1.598868 10659765

[48] Riley J Dehghani H Schweiger M Arridge SR Ripoll J Nieto-Vesperinas M. 3D Optical Tomography in the Presence of Void Regions. Opt Express (2000) 7(13):462–7. doi: 10.1364/oe.7.000462 19407898

[49] Lehtikangas O Tarvainen T. Hybrid Forward-Peaked-Scattering-Diffusion Approximations for Light Propagation in Turbid Media With Low-Scattering Regions. J Quant Spectrosc Radiat Transf (2013) 116:132–44. doi: 10.1016/j.jqsrt.2012.10.017

[50] Hull EL Foster TH. Steady-State Reflectance Spectroscopy in the P3 Approximation. JOSA A (2001) 18(3):584–99. doi: 10.1364/JOSAA.18.000584

[51] Shi Z Zhao H Xu K. Hybrid Diffusion-P3 Equation in N-Layered Turbid Media: Steady-State Domain. J BioMed Opt (2011) 16(10):105002. doi: 10.1117/1.3640810 22029346

[52] Yang D Chen X Cao X Wang J Liang J Tian J. Performance Investigation of SP 3 and Diffusion Approximation for Three-Dimensional Whole-Body Optical Imaging of Small Animals. Med Biol Eng Comput (2015) 53(9):805–14. doi: 10.1007/s11517-015-1293-8 25850985

[53] Wang L Cao H Cao X Ren S Li K Zhan Y. Adaptively Hybrid 3rd Simplified Spherical Harmonics With Diffusion Equation-Based Multispectral Cerenkov Luminescence Tomography. IEEE Access (2019) 7:160779–85. doi: 10.1109/ACCESS.2019.2950265

[54] Guo H Zhao H Yu J He X He X Song X. X-Ray Luminescence Computed Tomography Using a Hybrid Proton Propagation Model and Lasso-LSQR Algorithm. J Biophotonics (2021), e202100089. doi: 10.1002/jbio.202100089 34176239

[55] Liu J Wang Y Qu X Li X Ma X Han R. *In Vivo* Quantitative Bioluminescence Tomography Using Heterogeneous and Homogeneous Mouse Models. Opt Express (2010) 18(12):13102–13. doi: 10.1364/OE.18.013102 PMC2903618 20588440

[56] Gao Y Wang K An Y Jiang S Meng H Tian J. Nonmodel-Based Bioluminescence Tomography Using a Machine-Learning Reconstruction Strategy. Optica (2018) 5(11):1451–4. doi: 10.1364/OPTICA.5.001451

[57] Tian L Hunt B Bell MAL Yi J Smith JT Ochoa M. Deep Learning in Biomedical Optics. Lasers Surg Med (2021) 53(6):748–75. doi: 10.1002/lsm.23414 PMC8273152 34015146

[58] Li D Chen C Li J Yan Q. Reconstruction of Fluorescence Molecular Tomography Based on Graph Convolution Networks. J Opt (2020) 22(4):045602. doi: 10.1088/2040-8986/ab76a5

[59] Zhang Z Cai M Gao Y Shi X Zhang X Hu Z. A Novel Cerenkov Luminescence Tomography Approach Using Multilayer Fully Connected Neural Network. Phys Med Biol (2019) 64(24):245010. doi: 10.1088/1361-6560/ab5bb4 31770734

